# Draft Genome Sequence of *Acidobacteria* Group 1 *Acidipila* sp. Strain EB88, Isolated from Forest Soil

**DOI:** 10.1128/MRA.01464-18

**Published:** 2019-01-03

**Authors:** Luiz A. Domeignoz-Horta, Kristen M. DeAngelis, Grace Pold

**Affiliations:** a Department of Microbiology, University of Massachusetts, Amherst, Massachusetts, USA; b Graduate Program in Organismic and Evolutionary Biology, University of Massachusetts, Amherst, Massachusetts, USA; University of Arizona

## Abstract

Here, we present the genome sequence of a member of the group I *Acidobacteria*, Acidipila sp. strain EB88, which was isolated from temperate forest soil. Like many other members of its class, its genome contains evidence of the potential to utilize a broad range of sugars.

## Abstract (precis)

Here, we present the genome sequence of a member of the group I *Acidobacteria*, Acidipila sp. strain EB88, which was isolated from temperate forest soil.

## ANNOUNCEMENT

Group I *Acidobacteria* are abundant members of acidic-soil microbial communities (1), typically characterized by slow growth, heterotrophy, and the production of copious extracellular polysaccharides (2). Within this group, members of the genus Acidipila are characterized as acidophilic heterotrophs that use sugars as favored growth substrates (3, 4).

Acidipila sp. strain EB88 was isolated from the <0.8-μm size fraction of a deciduous forest mineral soil slurry plated onto VL45 plus glucose-yeast extract (GYE) medium (5). It was selected for sequencing based on the paucity of cultivated group I *Acidobacteria*, its peculiar bright-red waxy colonies on solid medium, and its undetectable growth in liquid medium unless a solid substrate, such as sand, is added.

DNA was prepared for sequencing by repeatedly freeze-thawing 9-day-old biomass scraped from pH 5 R2A medium and extracted using the Qiagen genomic DNA protocol. Sequencing at UMass Worcester using the PacBio RS II sequencer resulted in 476,266 filtered reads, with a mean length of 3,208 bp. The genome was assembled using sprai version 0.9.9.23 (http://zombie.cb.k.u-tokyo.ac.jp/sprai/index.html) and Canu version 1.5 (6). Default parameters were used for all tools unless otherwise specified. This yielded a 4.48-Mbp genome spanning 5 contigs, estimated as 100% complete and 0.86% contaminated using CheckM version 1.0.8 (7) in KBASE (8). Its GC content is 62.6%. Gene calling and annotation using the U.S. Department of Energy Joint Genome Institute’s annotation pipeline (9) identified 48 tRNA genes, 2 rRNA operons, and 3,593 protein-coding genes. TCDB (10) and TransAAP (11) annotated 392 of these genes as transporters.

The genome of EB88 is most closely related to the publicly available Acidipila rosea genome, with which it shares 94.5% 16S rRNA identity and 72.1% average nucleotide identity, according to IMG, and it has 60.9% mean amino acid identity using the average amino acid identity tool (http://enve-omics.ce.gatech.edu/aai/). The genome of EB88 is deficient in genes for organic acid uptake but contains genes for amino acid, ammonium, and nitrate uptake. It is capable of growth under both high- and low-oxygen conditions, and its genome contains five high-affinity *cbb*_3_ terminal oxidases and one low-affinity group A heme-copper oxygen oxidase (12). As is typical for the group I soil *Acidobacteria*, the genome of EB88 is rich in glycolytic enzymes and was found to contain 85 glycoside hydrolases in 48 families using HMMer searches in dbCAN (13). dbCAN also identified genes for capsule biosynthesis and export, including cellulose (14). However, rather than forming a copious hydrophilic capsule as is common for the group (2), EB88 has a waxy composition when grown on solid agar medium. It shares most of its lipid and fatty acid biosynthesis genes with other acidobacteria, so we posit that the hydrophobic nature of its colonies is driven by deficiencies in fatty acid degradation genes (EC 1.3.8.1, 2.3.1.9, and 2.3.1.16).

Together, these results indicate that the genome of Acidipila sp. EB88 is typical of many group I *Acidobacteria* but possibly differs in genes involved in extracellular associations. Its niche in forest soils is expected to include the use of sugars and amino acids under microaerobic to aerobic conditions.

### Data availability.

The complete genome sequence is available in GenBank under accession number QWEV00000000. The version described in this paper is the first version, QWEV01000000.
